# Integrating Artificial Intelligence in Pediatric Healthcare: Parental Perceptions and Ethical Implications

**DOI:** 10.3390/children11020240

**Published:** 2024-02-14

**Authors:** Elena Camelia Berghea, Marcela Daniela Ionescu, Radu Marian Gheorghiu, Iulia Florentina Tincu, Claudia Oana Cobilinschi, Mihai Craiu, Mihaela Bălgrădean, Florian Berghea

**Affiliations:** 1 “Marie S. Curie” Emergency Children’s Clinical Hospital, Carol Davila University of Medicine and Pharmacy, 041451 Bucharest, Romania; camelia.berghea@umfcd.ro (E.C.B.); mihaela.balgradean@umfcd.ro (M.B.); 2 National Institute for Mother and Child Health “Alessandrescu-Rusescu”, Carol Davila University of Medicine and Pharmacy, 041249 Bucharest, Romania; radu.gheorghiu@umfcd.ro; 3 Dr. Victor Gomoiu Clinical Children Hospital, Carol Davila University of Medicine and Pharmacy, 022102 Bucharest, Romania; iulia.tincu@umfcd.ro; 4 Sfanta Maria Clinica Hospital, Carol Davila University of Medicine and Pharmacy, 011172 Bucharest, Romania; claudia.cobilinschi@umfcd.ro (C.O.C.); florian.berghea@umfcd.ro (F.B.)

**Keywords:** artificial intelligence, medicine, pediatric, attitude, ethics

## Abstract

Background: Our study aimed to explore the way artificial intelligence (AI) utilization is perceived in pediatric medicine, examining its acceptance among patients (in this case represented by their adult parents), and identify the challenges it presents in order to understand the factors influencing its adoption in clinical settings. Methods: A structured questionnaire was applied to caregivers (parents or grandparents) of children who presented in tertiary pediatric clinics. Results: The most significant differentiations were identified in relation to the level of education (e.g., aversion to AI involvement was 22.2% among those with postgraduate degrees, 43.9% among those with university degrees, and 54.5% among those who only completed high school). The greatest fear among respondents regarding the medical use of AI was related to the possibility of errors occurring (70.1%). Conclusions: The general attitude toward the use of AI can be considered positive, provided that it remains human-supervised, and that the technology used is explained in detail by the physician. However, there were large differences among groups (mainly defined by education level) in the way AI is perceived and accepted.

## 1. Introduction

Artificial intelligence (AI), deep neural networks (DNN), and machine learning (ML) probably represent the fastest-developing technology area in recent years. AI, a branch of computer science, focuses on creating intelligent machines capable of performing tasks typically requiring human intelligence, including visual perception, speech recognition, decision-making, and language translation [1,2]. Widely used in almost all economic sectors, it was introduced from the very beginning into clinical, laboratory, or research medical activities. Although initially considered just another technology that could be applied to the medical world, it was quickly realized that AI has different characteristics from most other technologies due to its potential to replace human reasoning and even decision-making. As a result, debates quickly arose regarding its use, both in terms of the type of medical applications and the level of replacement of the human factor in the doctor–patient relationship. Interest in this topic saw a marked increase after the year 2021: internet searches for specific terms have increased nearly four-fold in recent years, reaching a maximum in April–May 2023 [3]. AI performs in areas such as fast adaptation, high diagnostic accuracy, and data management, which can help improve productivity. With this perceived potential, the FDA has continuously approved more machine learning (ML) software to be used by medical workers and scientists. However, there are also a few controversies, such as increased chances of data breaches, concern for clinical implementation, and potential healthcare dilemmas [4]. If the medical world approaches this technology in a way that is closer to the technical side, the patient partners receive extremely uneven information from sources of varying credibility [5]. Consequently, even in this new field, information inequality has manifested early, with the known consequences being a variable level of trust in the new technology and, consequently, an inequal degree of acceptance both at the level of patients and doctors [6,7]. The process of understanding and accepting AI is dynamic, driven, on one hand, by changes in the level of knowledge about the technology and, on the other, by its exponentially growing evolution. The result is a constant knowledge gap (between what this technology is and the way it is known) and an extremely dynamic change in the understanding of how it is accepted or even preferred compared to classical medical settings. Although there is a keen interest in understanding the mechanisms behind AI, the shared opinion, which we also endorse, is that people’s perception of AI depends more on contextual factors than on the underlying algorithms [5].

This topic has sparked particular interest in pediatrics, considering the way decisions are made for these patients (through delegating decisions to relatives, sometimes in an unequal manner) and the unique vulnerability of these patients, which adds extra pressure for faster access to medical decisions and a lower rate of errors. The evaluation of parental attitudes in these situations quickly gained the attention of researchers, leading to the crystallization of several areas of interest in the AI–patient relationship at this moment: quality/accuracy, privacy, shared decision-making, convenience, cost, the human element of care, and social justice [8,9].

Due to the extremely rapid developments in this field, we decided to undertake an exploration into the understanding of these attitudes at the current level of understanding and expansion of this technology. This study was conducted with the objective of understanding the attitude toward the use of AI among the relatives of children who use medical services.

## 2. Materials and Methods

We conducted a cross-sectional study in three tertiary pediatric units in Romania at the end of 2023. The study focused on assessing the knowledge and attitudes related to the current and potential use of AI in pediatric medicine. We used a locally developed, structured questionnaire following two focus groups (one including doctors and the other including relatives). We decided to redefine our questionnaire instead of using a pre-existing one due to the idea of adjusting this method according to scientific feedback after publication [9]. In developing the questionnaire, we started with similar, already published questionnaires (such as that by Sisk and colleagues [8]), which we filtered through comments and adjustments from later published research (for example, the study by Haley and colleagues, in which eight questions were changed due to interpretation difficulties [9]). The questionnaire was developed in Romanian. Three doctors conducted a focus group with five patients, starting with elements from studies that used validated or adapted questionnaires. Based on the preliminary conclusions, 22 questions were developed, which were later discussed in a group of 5 doctors who decided on some changes in the topical arrangement but did not alter the meaning of the questions. In the final evaluation, the study team considered that an additional question related to demographic information should be added. The final questionnaire (see Supplementary Materials) had 23 questions, making it easy to administer under the conditions of the study (which included parents of children who presented to the doctor, implying a high degree of nervousness and limited patience for such questions). Data collection included patients’ and parents’ age, parental education level, and yes/no answers for subjects’ awareness about AI. We were also interested in parental perception in terms of using AI in their child’s medical care, potential benefits and threats, and their opinion on informed consent before using AI. The questionnaire was entirely distributed in hard copy to the relatives of children who consecutively presented to the investigating doctors in the participating hospitals until we reached the target of 100 completed questionnaires. Based on the type of questionnaire (exploratory) and the data published in relevant situations, we considered that this number is capable of ensuring an acceptable level of robustness for the analysis. Subjects were identified in three major tertiary pediatric units, as follows: for each child aged 0–18 years who presented at these units during the study period, the questionnaire was administered to one accompanying person (parent or grandparent, in our case). There were no specific exclusion criteria other than those related to the respondents’ lack of desire to be interviewed or to sign the informed consent. Thus, all individuals were identified during a single week in the middle of December 2023. Statistical analysis was performed with the IBM SPSS Version 26.0 statistical software package. Data were expressed as mean ± standard deviation (SD). A value of *p* < 0.05 was considered statistically significant. The study did not involve clinical data, only sociological data, and was registered in December 2023 with the Research Ethics Committee (56/11 December 2023/3 January 2024/Sf. Maria Hospital). The inclusion of subjects was conditioned by the acceptance of informed consent obtained in the stage preceding the questionnaire administration.

## 3. Results

A small number of questionnaires (less than 10%) did not have responses to all questions, but these were also included in the final analysis. The sociodemographic data of the respondents are presented in Table 1 and the structure of the final questionnaire in Table 2.

**Figure 1:**
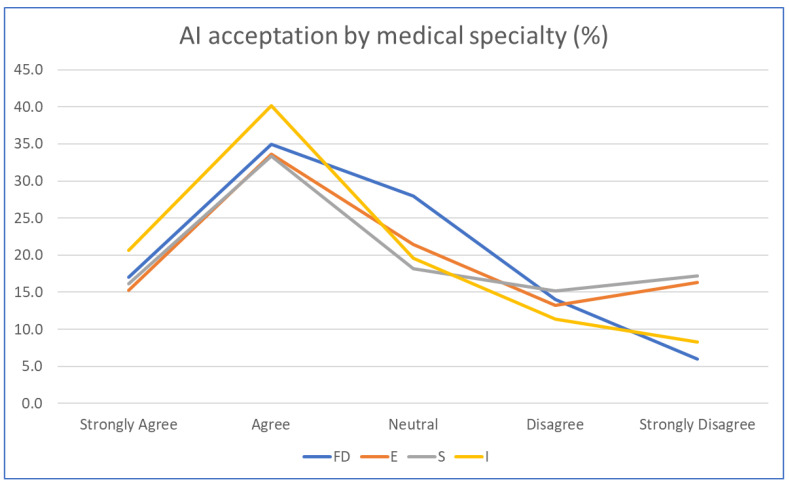
AI acceptance by medical specialty. FD: Family doctor; E: Emergency; S: Surgery; I: Imaging.

The vast majority (92%) of respondents declared that they have information about AI, but only 71% believed that AI is used in medicine (and another 20% said they were not sure about this). We found no significant difference from the point of view of education level between those who believed AI is used in medicine and those who did not (Student’s *t*-test, *p* = 0.143). A lower percentage considered that AI is used in their jurisdiction (29%), and only 7% believed that this had already happened in relation to their child. Additionally, there were no differences between the groups who believed that AI is already being used in their jurisdiction and those who disagreed with this, from the point of view of education level (the two groups were divided approximately equally at all levels of education). In terms of the field of AI use with which they would agree, we note that less than one-third of respondents strongly and very strongly opposed its use in any medical field. As can be seen in Figure 1, the most positive assessments occurred in the case of imaging, and the most reservations were in the case of surgery. We did not find relevant differences in the responses between men and women.

We have defined an AI adoption index in relation to medical specialties (*AIai*), computed as follows:

*AIai* = ∑*i* = 14*Ri*.

where *R*1 is the response in family medicine, *R*2 is the response in emergency medicine, *R*3 is the response in surgery, and *R*4 is the response in imaging. The value of *AIai* ranged between 4 (extremely positive attitude) and 20 (extremely negative attitude). We then monitored how this index correlated with the sociodemographic data: the education level and age of the parent. We observed a significant correlation between early adopters and education level (those favorable to a wider adoption had a higher level of education; Pearson correlation coefficient, r = −0.252; *p* 0.05). Analyzing only the responses of parents (excluding grandparents as relatives), the correlation proved to be stronger, with r = 0.304 at *p* < 0.01. At the same time, the age of the relative was not proven to be a determinant of the level of acceptance of AI in various medical specialties (r = 0.11). As expected, the gender of the child was not found as a determinant (Student’s *t*-test, *p* > 0.205). Judging by employment status and excluding grandparents, we found no statistically significant differences in terms of the AI adoption index among the three representative groups (medical professional, n = 15, non-medical professional, n = 56, and non-worker = 20), with index averages ranging from 10.37 to 11.25.

**Figure 2:**
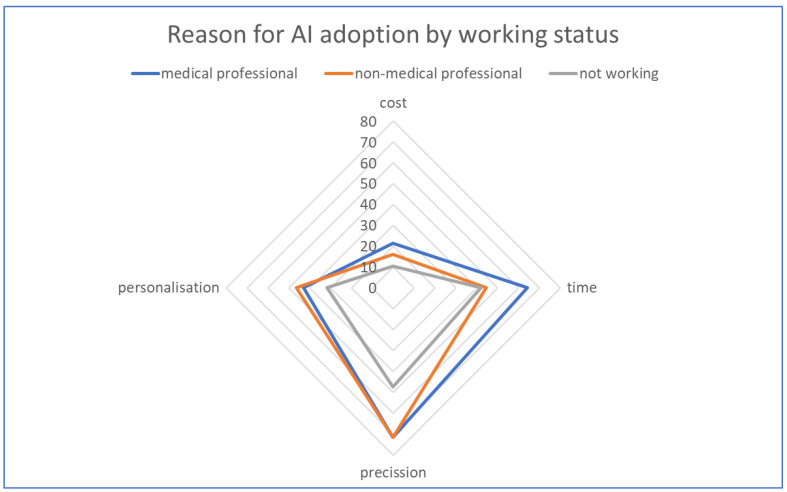
Reasons for AI adoption by working status.

When asked about how they prefer the therapeutic decision to be made (exclusively based on AI, exclusively on human reasoning, or a mix of the two), we observed an increase in aversion to AI involvement among groups with a lower level of education: 22.2% were in favor of exclusive use of human reasoning among those with postgraduate studies, 43.9% among those with university degrees, and 54.5% among those who only completed high school (the number of those with only primary education was too small to be analyzed here). On the other hand, regardless of the level of education, all subjects stated that it is important and very important for medical decisions to be supervised or reviewed by a human factor (where approximately 90% of respondents from all analyzed education groups considered this to be of utmost importance). Regarding the reasons that could lead to easier acceptance of AI use for their child, respondents ranked increased precision of medical decisions first (69.1%), followed by reduced waiting time and the possibility of better personalized therapy (46.4% and 43.3%, respectively), with the possibility of reducing medical expenses being ranked lowest (16.5%; Figure 2).

**Figure 3:**
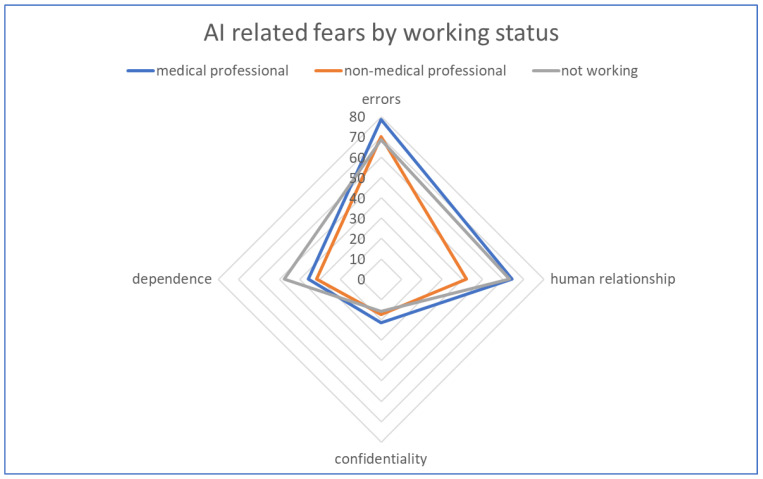
AI-related fears by working status.

The greatest fear among respondents regarding the medical use of AI was related to the possibility of errors occurring (70.1%), while the least concern was about the risk of losing the confidentiality of personal data (19.6%). At the same time, half of the respondents were concerned about losing their connection with the doctor in the case of extensive use of AI (Figure 3).

**Figure 4:**
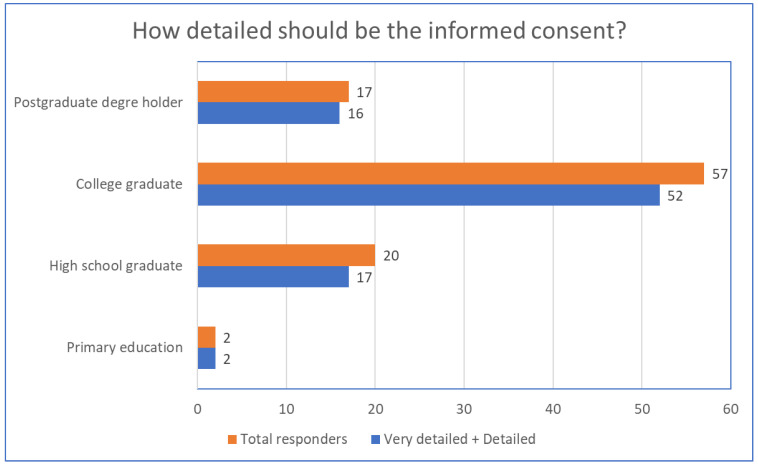
The amplitude of informed consent requested by the different caregiver education groups.

Regarding the necessity of obtaining parental consent before using AI, an impressive 88.8% of respondents considered this to be mandatory. As for the level of detail in the consent, 90.5% of respondents wanted extensive details about the use of AI, and only 5.2% agreed with a formal consent without detailed information. These proportions were found without significant differences across all groups, regardless of education level (Student’s *t*-test, *p* = 0.2; Figure 4).

Regarding information about the use of AI, 84% believed that this should be provided directly by the doctor, 27% agreed with individual informational materials (written or video), and 13% accepted information sessions in large groups. Regarding the final say in the decision to use or not use AI in the medical management of the child, the ratio between those who believed that the final decision should be the relatives, and those who believed the parent should be informed but, ultimately, the decision should be the doctors, changed as the education level increased (the overall value of this option was 57.1%). Thus, among the high school graduate respondents, 76.2% considered the decision to be exclusively the parent’s prerogative; then, the percentage dropped to 59.6% among those with a university education and reached 35.3% among those with postgraduate degrees. Moreover, in this last group, the proportion of those who believed the decision should be exclusively the doctors was the highest, at 11.8%.

Regarding the ethical aspects of AI use in medicine, we found that these were not widely considered: Only 17.5% of respondents believed that the introduction of AI could create inequality among various patients based on financial disparities or access to different medical resources. Slightly higher was the concern regarding the confidentiality of their child’s personal and medical data and how it is used (34.1%). These factors paled in comparison to the fear (re-assessed again in this round) that AI might produce errors (52.6%). The patient’s right to choose the use of AI was considered the most important right in relation to this technology, with respondents assigning it a score of 9.44 (1.62) on a scale from 0 to 10, where 10 is extremely important. A significantly lower value was obtained for the right to equal access to AI-assisted medical decisions: 8.6 (2.28).

## 4. Discussion

Applications of machine learning (ML) methods have been extensively used to solve various complex challenges in recent years in various application areas, such as medical, financial, environmental, marketing, security, and industrial applications [10].

AI currently represents a technology sector with one of the strongest growths, both in terms of intrinsic development and its adoption in various economic areas. In the healthcare sector, it is estimated that about 40% of the constituent industries are already using this technology [11]. From a market of approximately 20 billion USD, only in the USA, it is forecasted that in the next 5 years, this will reach a value of around 100 billion, and the global portable medical device industry is forecasted to attain more than 160 billion by 2033 [12], which implies plans to introduce this technology in the most diverse areas related to healthcare. Even now, AI is part of the healthcare system in ways in which the end consumer, the patient or their caregivers, perceive it more, or less. That is, AI is currently used in hospitals’ internal management to optimize resource allocation and hospital bed occupancy [13], or in identifying potential candidates and recruiting staff [14]. Closer to patient understanding is the area of medical equipment, which contains varying proportions of AI software elements. A 2021 study in the USA identified 222 medical devices and databases that contained AI elements (and 240 such items in Europe). However, the general public does not have a clear perception of how the use of these devices in their case actually marks the integration of AI into medical management, even though some of them have been classified as high-risk devices [15]. Patients have a clearer perception of potential interactions with AI when it comes to its use in various medical specialties, such as in medical imaging and in precision medicine [16,17,18]. Finally, the pharmaceutical industry, which fully utilizes this technology, should not be overlooked [19]. AI in medicine is expected to create a safe and equitable future, in the general context of an aging population and decreasing healthcare providers’ numbers and availability for prolonged on-site activity [20]. In the middle of the global nursing shortage, AI offers a solution to position patients’ needs with nursing expertise, informing equitable workload distribution [21].

AI-based systems have been explored in pediatric diagnosis for more than five years [22], with a high diagnostic accuracy across multiple organ systems and comparable performance to experienced pediatricians in diagnosing common childhood diseases. Even extremely sensitive areas, such as child abuse and domestic violence, have been approached by means of AI-driven instruments [23] via a retrospective screening tool, with a high accuracy of 0.90.

In high-impact areas, such as antibiotic prescribing in order to avoid antimicrobial resistance, machine learning has been used in recent years. A systematic review [24] that screened more than 3600 records included 5 high-quality studies, 2 of which were pediatric studies [25,26].

In severe diseases, a higher-than-average acceptance of tailored approaches has been proven. Patient-centered care delivery interventions, including patient-reported outcomes, hospital-at-home interventions, and other models of care, were developed for individuals with cancer. By demonstrating the relevance and utility of these different care models for patients with gastrointestinal malignancies, the authors of this literature review hoped to highlight the importance of developing and testing new interventions to address the unique needs of this population [27].

There have been extensively explored barriers and enablers for acceptance of AI innovations in a wide spectrum of medical fields. Most often, perception issues were approached in imaging tools, such as radiology [28] or oncology [29,30].

The ethics of AI implications in prenatal and genetic studies has also been researched [31] and was found to concur with concepts of beneficence, non-maleficence, respect for autonomy, justice, transparency, accountability, privacy, and trust.

The novelty of AI, along with the challenges related to the varying levels of understanding of the phenomenon, leads patients to desire increased control over it, whether we talk about personal control (knowledge, acceptance, or rejection) or institutional control (regulation, information, and transparency). The present study aimed to explore the attitudes of healthcare service consumers (represented here by individuals legally responsible for children treated in pediatric services), indirectly estimating the way this complex phenomenon is understood. There is a constant, repetitive effort to conduct such explorations, justified by the extremely rapid evolution of technology and its adoption in non-medical areas closer to consumer understanding. Specific studies suggest trends in AI acceptance and its positioning in relation to the human factor that justify a periodic refreshing of data [32].

Firstly, the study consistently showed an increased acceptance of AI across all medical fields, paralleling the rise in education level. Similarly, albeit at a different level, there was acceptance of decisions based solely on AI. However, our study indicated an insufficient understanding of how AI is already present in the medical field. We can surmise that it is precisely because of this lack of information that almost all subjects desired human supervision of AI decisions. This human supervision is, however, highly debatable when we talk about the automatic decision-making systems already incorporated into medical equipment (and in many cases, the users: technicians or doctors, are not aware of the presence of AI software). We noted that reducing medical costs was considered a minor objective in relation to the use of AI. This can be explained by the particular situation of health insurance in Romania: a low level of direct payments and 100% coverage of a wide range of medical services, including the entire pediatric healthcare system, which does not create particular pressure on personal budgets. This is an extremely delicate area for the future, as AI is seen by investors in healthcare services as a driver of cost reduction. Subjects mostly stated that they would wish to be informed about AI by their attending physician. This is a request that we can consider justified, but judging by the shortage of doctors, this is unlikely to happen (especially since the respondents preferred a more extensive explanation). Of course, this option may reveal a higher level of trust in medical personnel but also a lack of habit to self-inform from credible sources. We found that the education level of the respondents went hand-in-hand with the degree of freedom given to the doctor in choosing whether or not to use AI. We consider that such an attitude is more likely due to a broader context in which more educated individuals have more trust in the decisions of doctors, whether these refer to AI (as a technology) or something else. It remains to be understood why the issue of personal and medical data confidentiality was not considered of utmost importance by the respondents. We can presume that this is due to a lack of understanding of the importance of the subject, or alternatively because the relative importance of this subject compared to the main therapeutic objective is indeed lower.

Although we found differences between respondents with a medical background and those from other fields, it must be considered that within the medical community, there is a variable perception and acceptance of AI. Furthermore, elements of medical priority (reducing costs, reducing time, improving accuracy, etc.) are not equally recognized as valuable by those who work in the medical field and those who do not.

Previous studies have shown similar results in areas also found in our study. For example, a study conducted in the UK in 2021 with 408 patients noted that less than 36% of the respondents were aware of the existence and utility of AI [17]. Another study in Germany in 2022 identified only 24% of patients in this situation. It is interesting that many of the conclusions of this study also appeared in our setting (which, unlike the others, refers to the patients’ parents, not the patients themselves): there was a predominant desire for the final decision to belong to the doctor and not AI, responsibility should lie with the doctor, and the doctor must be aware of the limits of AI. Both mentioned studies revealed a relatively low interest in the risk of incorrect use of patient personal data, similar to our results [33].

The strength of this study comes from its benefits from a broader perspective on the phenomenon of understanding and accepting AI in pediatrics by including subjects from three independent tertiary clinical units. Another advantage is the very short duration of its execution, allowing a true cross-sectional view through the study population at this moment (important considering the speed at which information about AI emerges). Additionally, based on comments regarding similar studies conducted in the past, we were able to adjust the questionnaire to cover previously identified gaps. The study was conducted in Romania, a country characterized in the last 10 years by an accelerated dynamic of economic growth compared to Western European countries, dynamics largely based on adopting new information technologies. Even before the COVID-19 pandemic, Romanian citizens confronted with health problems were prone to first access online resources and self-medication. In 2017, a local study documented that 32% of respondents took medicines after consulting a website [34]. This proportion has remained elevated in recent years, and in a 2020 paper it was documented that parents used self-medication for their children, 61% sometimes and 9% frequently [35]. A national evaluation in 2021—the Public Health Barometer—documented that 25% of Romanians used only self-accessed online resources for healthcare [36]. In this context, the Romanian population could be more open to this type of change due to a perception bias. Patients have attitudes and behavior patterns related to risk-perception and they tended to approach self-medication even before the pandemic [37]. Online and AI-driven algorithms do function [38], and Romanian parents have an increased appetite of such tools because of the IKEA effect [39]. In addition, AI chatbots have proven effective in health behavior change interventions across broad and varied populations [40].

The COVID-19 pandemic introduced a new paradigm of healthcare in Romania: telemedicine and telehealth [41]. In Romania, telemedicine was formally implemented starting September 2022, so the current findings should be evaluated in the context of an increasing usage of tools that are incorporating AI and algorithms in healthcare [42].

The study’s limitations are due to the poor inclusion of subjects with low education levels (due to the mechanism of consecutive introduction of patients presented to investigators), the lack of structured information material for respondents about what AI in medicine means, and the listing and explanation of ethical aspects. The number of subjects could be considered another limitation. However, due to the informational explosion related to AI (where practically every day there is at least one reference to AI in mass media or social media), we decided to be extremely limited in the duration of the study (one week). Even if this might have implications for the robustness of the data, we believe that we are in a very fluid period regarding the perception of AI in medicine and, therefore, it was more important to capture a characteristic of the moment and possibly, by repeating the study, to identify a trend.

## 5. Conclusions

The present study evaluated the attitude of the relatives of children who attended three tertiary medical services regarding the use of artificial intelligence in their child’s case. The degree of acceptance increased concordantly with the education level of the respondents but, for the present scenario in Romania, there is a significant need to improve the level of knowledge about AI’s potential in medicine. Because a fear of AI errors prevails, a transparent and solid regulatory framework should be assembled. A potential strategy to deploy such practical tools in clinical medicine could be represented by a shared AI–human decision-making process. Such a paradigm must be implemented, at least for the initial steps of such an endeavor.

Targeting parents via simple and accessible communication tools, such as social media, could decrease hesitancy and increase adoption of AI-driven protocols, both in research and clinical management of disease. Similar studies could be repeated on a regular basis in order to capture the social trends in perception.

## Figures and Tables

**Table 1:** Demographic data.

Age of the relative (years)—Mean (SD)	37.5 (8.05)	N = 98
Age of the child (years)—Mean (SD)	6.63 (3.88)	N = 98
Gender of the child—No. of valid answers		N = 100
Male	33%	
Female	67%	
Education level of the respondent—No. of valid answers		N = 99
Primary education	2%	
High school graduate	22.2%	
College graduate	57.6%	
Postgraduate degree holder	18.2%	
Employment status of the respondent—No. of valid answers		N = 99
Student	0%	
Medical professional	14.2%	
Non-medical professional	59.6%	
Retired	3%	
Other	23.2%	

N = number of subjects.

**Table 2:** Structure of the final questionnaire.

Area	Number of Questions
Demographic data	5
Familiarization with the term AI	1
Knowledge of AI utilization in medicine	3
Use of AI in different medical fields	4
Acceptance of AI in the context of childcare	2
Fears regarding the use of AI in medicine	2
The need for parent information and obtaining informed consent prior to the use of AI	4
Ethics of AI usage	2

## Data Availability

Data are contained within the article.

## Supplementary Materials

The following supporting information can be downloaded at: https://www.mdpi.com/article/10.3390/children11020240/s1.
