# Supplementary material for: Integrating Artificial Intelligence in Pediatric Healthcare: Parental Perceptions and Ethical Implications
Source: Children (Basel). 2024 Feb 14;11(2):240. doi: 10.3390/children11020240 (PMC10887612; doi:10.3390/children11020240)
Supplement: Supplementary file 1 [file children-11-00240-s001.zip › children-2827076-supplementary.pdf]

Supplementary Materials – The final questionnaire

1A. Your age is ………. years    1A. The age of YOUR CHILD is ………. years

2. Gender: (CHECK THE CORRECT BOX)

◯ Male ◯ Female ◯ Other/Prefer not to answer

3. Occupation:

◯ Student ◯ Healthcare Professional ◯ Professional in another field ◯ Retired

◯ Other

4. Education level:

◯ Elementary School ◯ High School ◯ University ◯ Postgraduate

5. Have you heard of the term "artificial intelligence" (AI)? (CHECK THE CORRECT BOX)

◯ Yes ◯ No

6. Do you believe AI is used in medicine?

◯ Yes ◯ No ◯ Not sure

7. Do you believe AI is used in medicine IN ROMANIA?

◯ Yes ◯ No ◯ Not sure

8. Do you believe AI has ALREADY been used in medicine for your child?

◯ Yes ◯ No ◯ Not sure

9. To what extent do you agree with the use of AI by YOUR CHILD'S FAMILY DOCTOR?

◯ Strongly agree ◯ Agree ◯ Neutral ◯ Disagree ◯ Strongly disagree

10. To what extent do you agree with the use of AI FOR EMERGENCY SITUATIONS FOR YOUR CHILD?

◯ Strongly agree ◯ Agree ◯ Neutral ◯ Disagree ◯ Strongly disagree

11. To what extent do you agree with the use of AI in SURGERY FOR YOUR CHILD?

◯ Strongly agree ◯ Agree ◯ Neutral ◯ Disagree ◯ Strongly disagree

12. To what extent do you agree with the use of AI IN RADIOLOGY OR MRI FOR YOUR CHILD?

◯ Strongly agree ◯ Agree ◯ Neutral ◯ Disagree ◯ Strongly disagree

13. To what extent do you prefer medical decisions based solely on AI compared to those based on human reasoning?

◯ Prefer MEDICAL DECISIONS BASED solely on AI

◯ Prefer MEDICAL DECISIONS BASED on a mix of AI and my doctor's reasoning

◯ Prefer MEDICAL DECISIONS BASED solely on my doctor's reasoning

14. Would you be more accepting of the use of AI in medicine if it resulted in: (check as many options as relevant)

◯ Lower costs for me

◯ Shorter waiting times and faster appointments with the doctor

◯ Higher accuracy in diagnosis

◯ Personalized treatments

◯ Other (please specify)______________________________

15. What are your main concerns regarding the use of AI in medicine? (Select all relevant options)

◯ AI can make errors

◯ Using AI loses the human connection with the doctor

◯ The use of AI will lead to the loss of confidentiality of my personal data

◯ The use of AI will create excessive dependence on technology

◯ Other (please specify)

16. How important is it to you that medical decisions are supervised or reviewed by a human doctor?

◯ Very important ◯ Important ◯ Neutral ◯ Slightly important ◯ Not important at all

17. Do you believe parental consent is necessary for the use of AI in the diagnosis or treatment of YOUR CHILD?

◯ Yes, always

◯ Only in life or death decisions

◯ No, it is not necessary

18. In case parental consent is required for the use of AI, how much information should this consent include?

◯ A VERY detailed consent, including a description of the AI process, benefits, and potential risks

◯ A DETAILED BUT SHORTER consent, presenting the benefits and general use of AI

◯ A VERY SHORT consent, mentioning only that AI will be used in treatment

◯ I have no formed opinion

19. How should parental information about the use of AI in their CHILD'S medical care be approached?

◯ Through detailed discussions with the doctor

◯ Through written or digital informative materials

◯ Through counseling sessions or educational seminars

◯ Other methods (please specify)

20. To what extent should PARENTS be able to refuse the use of AI in their CHILD'S treatment?

◯ Completely - PARENTS should have complete control

◯ Partially - PARENTS should have a SAY BUT IN THE END THE DOCTOR WILL DECIDE

◯ Not at all - decisions should be made exclusively by doctors

◯ Not sure

21. What are your main ethical concerns regarding the use of AI in medicine? (Select all relevant options)

◯ With AI, you can no longer be sure where your child's medical information goes and who uses it

◯ With AI, you can no longer be sure that medical decisions are fair and the same for everyone regardless of wealth or connections

◯ With AI, you can no longer understand who decides for your child and why a certain decision was made

◯ With AI, there is an increased risk of a machine making a wrong decision for your child

◯ With AI, you no longer have the same human connection with your doctor

◯ Other concerns (please specify)

22. How important are the following aspects related to the ethics of AI usage in medicine to you? (RATE ON A SCALE FROM 0 – NOT IMPORTANT TO 10 – EXTREMELY IMPORTANT)

Informed consent …………….

Patient's right to choose…………..

Equality of access to AI-assisted treatments…………

Accountability for AI errors……….
